# Kontrazeption für den Mann – Wissenschaftliche Erkenntnisse und Entwicklungen

**DOI:** 10.1007/s00103-026-04208-8

**Published:** 2026-02-26

**Authors:** Michael Zitzmann

**Affiliations:** Centrum für Reproduktionsmedizin und Andrologie, Klinische Andrologie, Universitätsklinikum Münster, Domagkstr. 11, 48149 Münster, Deutschland

**Keywords:** Männliche Kontrazeption, Hormonelle Kontrazeption, Suppression der Spermatogenese, Nichthormonelle Kontrazeption, Männliche reproduktive Gesundheit, Male contraception, Hormonal contraception, Suppression of spermatogenesis, Non-hormonal contraception, Male reproductive health

## Abstract

Trotz jahrzehntelanger Forschung und gesellschaftlichen Interesses sind die Verhütungsoptionen für den Mann stark eingeschränkt. Seit Frauen von hormonellen Methoden profitieren, liegt die Hauptverantwortung für Kontrazeption nahezu ausschließlich bei ihnen. Angesichts möglicher Nebenwirkungen weiblicher Hormonpräparate und des Rechts auf körperliche Selbstbestimmung rückt die Entwicklung sicherer, reversibler Methoden für Männer in den Fokus.

Die hormonelle Kontrazeption beim Mann basiert auf der Suppression der Spermienbildung durch exogenes Testosteron in Kombination mit Gestagenen. Multicenterstudien mit Testosteronundecanoat und Norethisteron-Enantat zeigten eine kontrazeptive Wirksamkeit, die oralen Kontrazeptiva der Frau entspricht. Nebenwirkungen wie Libidoverlust, depressive Verstimmungen und Gewichtszunahme führten zum Abbruch der World Health Organization (WHO)-geführten Studie. Transdermale Gelkombinationen (Testosteron/Nestoron) und neue orale Wirkstoffe zeigen ein günstigeres Verträglichkeitsprofil.

Parallel gewinnen nichthormonelle Ansätze an Bedeutung. Dazu zählen Retinsäure-Rezeptor-(RARα‑)Antagonisten wie YCT-529, die die Spermatogenese gezielt hemmen, sowie Inhibitoren der löslichen Adenylatzyklase (sAC), die eine bedarfsgerechte Kontrazeption durch reversible Hemmung der Spermienmotilität ermöglichen. Beide Strategien erwiesen sich in präklinischen Studien als hochwirksam, reversibel und gut verträglich; YCT-529 befindet sich in klinischer Erprobung.

Die Entwicklung männlicher Kontrazeptiva wird aktuell von akademischen und gemeinnützigen Institutionen vorangetrieben. Obwohl marktreife Präparate noch ausstehen, verdeutlichen die Fortschritte in hormonellen und nichthormonellen Ansätzen das Potenzial für eine gerechtere Verteilung der Verhütungsverantwortung.

## Abstract (en)

Despite decades of assiduous inquiry and burgeoning societal demand, contraceptive options for men remain circumscribed. Women have benefitted from highly efficacious hormonal modalities, but the burden of contraceptive responsibility continues to rest almost exclusively upon them. In light of the potential sequelae of female hormonal agents and the inviolable right to bodily autonomy, the quest for safe, reversible male methods is assuming urgency.

Hormonal contraception for men relies on suppression of spermatogenesis through exogenous testosterone combined with progestins. Multicenter trials employing testosterone undecanoate with norethisterone enanthate demonstrated contraceptive efficacy commensurate with oral contraceptives in women. Nonetheless, adverse sequelae—including diminished libido, dysphoric mood, and weight accrual—precipitated discontinuation of the WHO-led trial. Recent transdermal combinations (testosterone with nestorone) and novel oral androgens have exhibited encouraging tolerability.

Concurrently, non-hormonal paradigms are garnering momentum. Retinoic acid receptor‑α antagonists such as YCT-529 disrupt spermatogenic differentiation, while inhibitors of soluble adenylyl cyclase transiently impair sperm motility, enabling on-demand, pharmacological fertility control. Both strategies have proven efficacious, reversible, and well tolerated in preclinical investigations; notably, YCT-529 has advanced into human trials.

At present, the vanguard of innovation lies with academic consortia and philanthropic institutions rather than industry. Although a market-ready preparation remains elusive, the progress in both hormonal and non-hormonal avenues underscores the profound potential for a more equitable apportionment of contraceptive responsibility.

## Einleitung

Trotz jahrzehntelanger Forschung und eines gesellschaftlichen Interesses sind die Verhütungsoptionen für den Mann stark begrenzt. Während die gesellschaftliche und medizinische Wahrnehmung hormoneller Kontrazeptiva für Frauen gegenwärtig einem kritischen Wandel unterliegt, bleibt Männern nach wie vor nur ein sehr eingeschränktes Spektrum an Methoden zur Verfügung. Zunehmend wird deutlich, dass hormonelle Kontrazeptiva für Frauen nicht nur Vorteile, sondern auch erhebliche körperliche und seelische Belastungen mit sich bringen. Vor diesem Hintergrund wächst die Forderung, dass die Verantwortung für Kontrazeption nicht einseitig bei den Frauen liegen darf.

Dieser Paradigmenwechsel findet sich sowohl im öffentlichen Diskurs als auch in internationalen gesundheitspolitischen Foren wieder, wo verstärkt betont wird, dass Männer nicht nur von den Errungenschaften moderner kontrazeptiver Technologien profitieren, sondern auch bereit sein sollten, Verantwortung für deren Anwendung zu übernehmen. Dieses Verständnis steht in engem Zusammenhang mit dem Recht der Frau auf körperliche Unversehrtheit, zumal weltweit jährlich mehrere Hunderttausend Frauen infolge unsachgemäß durchgeführter Abtreibungen sterben [1]. Parallel hierzu wächst bei Männern das Interesse, ihre reproduktive Gesundheit selbst zu gestalten und die Kontrolle über ihre Fertilität nicht der Partnerin zu überlassen. 80–90 % der Männer wären bereit, neue Verhütungsmethoden anzuwenden [2, 3]. Ebenso lassen die Daten erkennen, dass Frauen der Zuverlässigkeit und korrekten Anwendung männlicher Kontrazeptiva durch ihre Partner überwiegend vertrauen [4].

Es besteht somit eine gesellschaftliche Nachfrage nach wirksamen, sicheren und reversiblen Kontrazeptiva für den Mann. Dass trotz langer Forschung bislang keine solche Option in die medizinische Versorgung eingeführt wurde, verweist auf erhebliche wissenschaftliche, ökonomische und gesellschaftliche Hürden. Ziel dieses Beitrags ist es daher, die biologischen, pharmakologischen und klinischen Dimensionen männlicher Kontrazeption darzustellen, aktuelle Forschungsansätze kritisch zu würdigen und sie zugleich im größeren Kontext gesellschaftlicher Verantwortung sowie ethischer Fragestellungen zu verorten.

Zum Zwecke der Transparenz sei darauf hingewiesen, dass ein Teil der hier dargestellten Inhalte bereits in dem Beitrag „Verhütung beim Mann – aktueller Stand“ vor Kurzem erörtert wurde [5]. Der vorliegende Artikel geht jedoch deutlich darüber hinaus, indem er erstmals einen umfassenden Überblick über nichthormonelle Methoden der männlichen Kontrazeption bietet und zudem die ethisch-philosophischen Dimensionen der Kontrazeptionsforschung im Kontext veränderter Geschlechterrollen vertieft beleuchtet.

## Ethische Perspektive auf Kontrazeptionsgleichheit

Die Frage nach der Gleichverteilung kontrazeptiver Verantwortung berührt nicht allein medizinische Praktikabilität, sondern das Fundament anthropologischer und ethischer Selbstverwirklichung. Es ließe sich argumentieren, dass die Pflicht zur gegenseitigen Achtung der Autonomie verlangt, dass weder Mann noch Frau einseitig die Last reproduktiver Verantwortung zu tragen habe. So erweist sich die Kontrazeption als konkretes Feld der Anwendung der Maxime, den Mitmenschen niemals bloß als Mittel eigener Absichten zu gebrauchen, sondern ihn in jedem Fall als Zweck und Sinn an sich zu respektieren.

Man könnte in der Entwicklung männlicher Kontrazeptiva einen Fortschritt des sittlichen Lebens erkennen: Die Anerkennung des anderen im gemeinsamen Projekt der Familienplanung erweitert die Freiheit beider Geschlechter. In der Dialektik von Subjektivität und Objektivität wird Kontrazeption nicht mehr nur als technisches Mittel, sondern als Ausdruck wechselseitiger Anerkennung und freier Vernunftpraxis verstanden.

Schließlich rückt das „In-der-Welt-Sein“ des Menschen in den Fokus: Reproduktion und ihre Begrenzung sind nicht bloß biologische Vorgänge, sondern existenziale Entwürfe, in denen sich Freiheit, Sorge und Verantwortung artikulieren. In dieser Perspektive verweist die Entwicklung männlicher Kontrazeption auf die Möglichkeit, dem eigenen Dasein eine neue Weise des „Fürsorge-Seins“ zu geben – nicht nur für sich, sondern im Mitsein mit dem anderen.

So gesehen ist die medizinische Aufgabe der kontrazeptiven Gleichstellung mehr als eine technische Innovation: Sie wird zu einem Ausdruck praktischer Vernunft, sozialer Anerkennung und existenzieller Fürsorge, die über das Biologische hinaus in die Sphäre des Ethischen und Humanen hineinragt.

## Zentrale Begriffe und Anforderungen an eine Kontrazeptionsmethode

Die Diskussion männlicher Kontrazeption setzt zunächst ein Verständnis biologischer Grundlagen voraus. Unter Spermatogenese versteht man den Prozess der Entwicklung von Spermien. Ein vollständiges Fehlen von Spermien im Ejakulat wird als Azoospermie bezeichnet und ist häufig das erwünschte Endziel einer wirksamen Kontrazeptionsmethode. Die Steuerung dieser Prozesse erfolgt maßgeblich durch Gonadotropine, namentlich das follikelstimulierende Hormon (FSH) und das luteinisierende Hormon (LH), die in der Hypophyse gebildet werden und die Funktion der Hoden regulieren.

Die Entwicklung einer pharmakologischen Methode zur männlichen Kontrazeption unterliegt hohen Anforderungen, die über die gängigen Kriterien therapeutischer Arzneimittel hinausgehen. Während bei Krankheiten Nebenwirkungen in Kauf genommen werden können, weil der Nutzen überwiegt, ist die Ausgangslage hier anders: Gesunde Männer würden sich freiwillig und über lange Zeit einem Wirkstoff aussetzen – nicht zur eigenen Heilung, sondern ausschließlich, um eine Schwangerschaft bei der Partnerin zu verhindern. Eine solche Selbstverpflichtung ist nur dann legitim, wenn sie höchsten Standards von Sicherheit und Verträglichkeit genügt.

Daraus ergeben sich mehrere Kriterien: Eine männliche Verhütungsmethode darf das körperliche und geistige Wohlbefinden nicht beeinträchtigen und muss insbesondere die sexuelle Funktion unangetastet lassen. Ebenso ist ein definierter Wirkungseintritt erforderlich, die Wirksamkeit muss den etablierten weiblichen Methoden entsprechen und die Reversibilität gilt als unverzichtbar, um eine spätere Vaterschaft zu ermöglichen. Darüber hinaus muss die Sicherheit potenzieller Nachkommen garantiert sein – teratogene oder genetische Schäden wären inakzeptabel.

Im Lichte philosophischer Reflexion gewinnt diese Sicherheitsforderung eine noch tiefere Dimension. Kontrazeption darf niemals riskieren, den Mann oder seine potenziellen Nachkommen bloß als Mittel zu instrumentalisieren; sie ist nur dann moralisch vertretbar, wenn sie die Würde und Autonomie aller Beteiligten respektiert. Freiheit zeigt sich hier als gegenseitige Anerkennung innerhalb der Partnerschaft: Indem Männer Verantwortung für Verhütung übernehmen, gleichen sie ein historisches Ungleichgewicht aus und schaffen ein neues Verhältnis partnerschaftlicher Gleichberechtigung. Der Einsatz einer solchen Methode wäre daher nicht nur Ausdruck biomedizinischer Innovation, sondern auch ein ethisch-politischer Akt, in dem Verantwortung, Gerechtigkeit und Fürsorge untrennbar ineinanderfließen.

## Wirkmechanismus der hormonellen Kontrazeption

Die Entwicklung hormoneller Methoden zur männlichen Verhütung nahm ihren Anfang mit dem Erfolg oraler Kontrazeptiva für Frauen. Trotz unterschiedlicher Entwicklungswege beruht das Prinzip auf einem gemeinsamen biologischen Mechanismus: der gezielten Modulation der endokrinen Rückkopplung zwischen Hypothalamus, Hypophyse und Hoden. Diese Achse ist das zentrale Steuerungssystem der männlichen Fertilität.

Unter physiologischen Bedingungen regulieren Hypophyse und Hoden die Spermienbildung in enger Abstimmung. Das follikelstimulierende Hormon (FSH) aktiviert die Sertoli-Zellen in den Samenkanälchen und ermöglicht damit die Reifung der Keimzellen. Gleichzeitig stimuliert das luteinisierende Hormon (LH) die Leydig-Zellen, Testosteron zu produzieren, das intratestikulär unverzichtbar für die Aufrechterhaltung der Spermatogenese bleibt. Aus Stammzellen der Keimbahn entwickeln sich über eine Abfolge komplexer Schritte die Spermien. Das Ziel hormoneller Kontrazeption ist es, diesen Prozess bis hin zur Azoospermie – dem völligen Fehlen von Spermien im Ejakulat – zu unterdrücken.

**Abb. 1:**
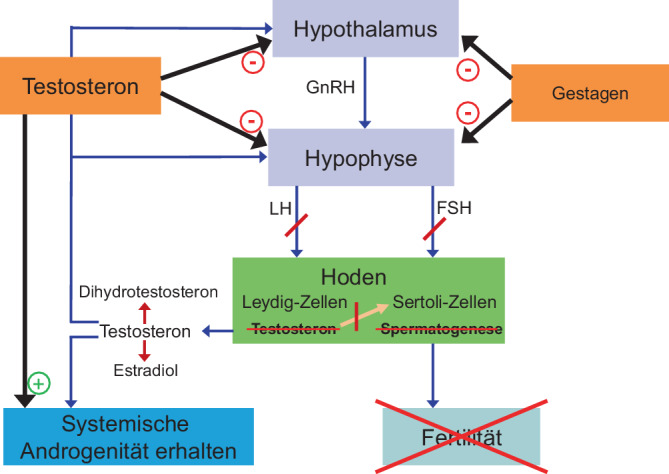
Prinzip der hormonellen Kontrazeption beim Mann. Schematische Darstellung der Rückkopplungsschleife zwischen Hypothalamus, Hypophyse und Hoden sowie der hemmenden Effekte von exogenem Testosteron und einem Gestagen auf die Hodenfunktion, begleitet von der Substitution der Testosteronwirkung im Rest des Körpers. Das durchgestrichene Wort „Testosteron“ im Hoden soll veranschaulichen, dass unter der Gabe von hormonellen Kontrazeptiva kein eigenes Testosteron mehr im Hoden hergestellt wird. Da aber von außen Testosteron zugeführt wird (eben als Teil des Kontrazeptivums) ist dies nur insofern relevant, als dass die natürliche Testosteronwirkung von dem eingesetzten Präparat möglichst widergespiegelt werden muss (also nicht zu hohe oder zu niedrige Testosteronspiegel durch das Kontrazeptivum sind anzustreben). Dihydrotestosteron entsteht durch 5‑alpha-Reduktion von Testosteron und bindet sehr stark an den Androgenrezeptor. Die Wirkung ist in Zielgeweben (z. B. Prostata, Haut/Haarfollikel, Gehirn (als Neurotransmitter)) besonders ausgeprägt. Estradiol entsteht durch das Enzym Aromatase aus Testosteron und ist beim Mann besonders für die Knochengesundheit, aber auch das psychische Wohlbefinden essenziell. Das natürliche Testosteron und zugelassene Testosteronpräparate werden auf diese Weise umgewandelt und das ist auch essenziell. Ein Androgen, das für die Verhütung eingesetzt wird, sollte ebenfalls in Dihydrotestosteron und auch Estradiol umgewandelt werden können, um die notwendige Wirkung im Körper entfalten zu können und den Steroidmetabolismus in der Balance zu halten. *FSH* follikelstimulierendes Hormon; *GnRH* Gonadotropin-Releasing-Hormon; *LH* luteinisierendes Hormon; Quelle: eigene Abbildung. Das Copyright liegt beim Autor (M. Zitzmann)

Würde man die endogene Testosteron-Produktion jedoch ohne Ersatz ausschalten, entstünde ein hypogonadaler Zustand mit klinisch relevanten Symptomen: Libidoverlust, Stimmungsschwankungen, Verlust an Muskel- und Knochenmasse. Daher gilt die Gabe von exogenem Testosteron als Grundpfeiler hormoneller Strategien. Das zugeführte Testosteron unterdrückt die Ausschüttung des Gonadotropin-Releasing-Hormons (GnRH) und damit die Freisetzung von LH und FSH. In den meisten Fällen reicht Testosteron allein jedoch nicht aus, weshalb zusätzlich ein Gestagen verabreicht wird, das die Gonadotropine weiter dämpft und so die Spermatogenese effektiv unterbindet (Abb. 1).

Da oral gegebenes Testosteron durch den hepatischen First-Pass-Metabolismus unwirksam wird, müssen alternative Wege gewählt werden: intramuskuläre Injektionen, subkutane Implantate oder transdermale Systeme wie Testosteron-Gele.

In ethischer Reflexion würde dies bedeuten, dass die hormonelle Kontrazeption beim Mann einen gesunden Organismus gezielt in einen Zustand pharmakologisch induzierter Keimzellunterdrückung versetzt. Dies wirft grundlegende Fragen auf: Ist es gerechtfertigt, physiologische Funktionen bewusst außer Kraft zu setzen, wenn dadurch eine Schwangerschaft verhindert werden kann? Es ließe sich argumentieren, dass der Mann hier nicht nur seine eigene Autonomie ausübt, sondern zugleich seine Partnerin als gleichwertigen Menschen anerkennt. Er übernimmt durch die hormonelle Verhütung Verantwortung, um ihre Belastung durch hormonelle Eingriffe zu reduzieren.

Auf gesellschaftlicher Ebene wird die männliche Kontrazeption damit zu einer Frage geteilter Verantwortung. Sie entzieht sich einer rein medizinischen Diskussion und wird zu einem Spiegel gesellschaftlicher Normen und Rollenbilder: Wer trägt die Last der Verhütung? Wer ist bereit, Nebenwirkungen auf sich zu nehmen, um den anderen zu entlasten? In diesem Sinne ist die pharmakologische Unterdrückung der Spermatogenese nicht nur ein biomedizinischer Mechanismus, sondern zugleich ein ethisch-politischer Akt – Ausdruck von Partnerschaft, Gleichberechtigung und geteilter Verantwortung**.**

## Studien zu Wirksamkeit, Reversibilität und Nebenwirkungen: Historie und aktueller Stand

Frühe klinische Studien zur männlichen hormonellen Kontrazeption dienten dem Machbarkeitsnachweis. In jüngerer Zeit wurden größere multizentrische Studien durchgeführt, die vor allem lang wirksame injizierbare Testosteronpräparate wie Testosteronundecanoat prüften. Damit konnte erstmals eine differenziertere Bewertung von Wirksamkeit, Reversibilität und Nebenwirkungen erfolgen. Verschiedene Gestagene wurden in Kombination mit Testosteron verabreicht. Ziel war stets eine weitgehende Unterdrückung der Gonadotropine zur verlässlichen kontrazeptiven Wirkung [6–9].

Eine placebokontrollierte Studie bestätigte typische Nebenwirkungen bei etwa 10 % der Teilnehmer: nächtliches Schwitzen, verminderte Libido und moderater Gewichtsanstieg [10]. Diese Effekte werden mit proinflammatorischen und antiandrogenen Eigenschaften bestimmter Gestagene erklärt [11]. Insgesamt wurde die Verträglichkeit jedoch als akzeptabel bewertet.

Bislang fehlen jedoch systematische Langzeitdaten. Die meisten Studien waren auf höchstens 12 Monate begrenzt, sodass mögliche kardiovaskuläre, metabolische oder reproduktive Risiken nicht abschließend beurteilt werden können.

Gerade weil es sich um gesunde Probanden handelt, die ein Medikament nicht zur Therapie, sondern allein zur Verhütung einnehmen, ist das Fehlen von Langzeitdaten besonders kritisch. Aus ethischer Sicht gilt: Eine Methode, die freiwillig von Gesunden angewandt wird, muss einen Sicherheitsstandard erfüllen, der über dem therapeutisch Notwendigen liegt. Solange belastbare Langzeitergebnisse fehlen, bleibt die Einführung solcher Präparate in die klinische Routine möglicherweise moralisch fragwürdig.

Eine bedeutende klinische Studie zur männlichen hormonellen Kontrazeption wurde in China durchgeführt: Über 1000 Paare nahmen daran teil, wobei Testosteronundecanoat als Monotherapie – ohne Zusatz eines Gestagens – geprüft wurde. Unter diesen Bedingungen konnte eine zuverlässige Azoospermie erreicht werden, die sich in einem Pearl-Index von 1,1 niederschlug – einem Wert, der der kontrazeptiven Sicherheit oraler Pillen bei Frauen entspricht [12]. Für europäische Männer sind jedoch meist Kombinationen aus Testosteron und Gestagen erforderlich, um eine vergleichbare Wirksamkeit zu erzielen. Diese Befunde verdeutlichen sowohl das Potenzial als auch die populationsspezifischen Grenzen einer Monotherapie.

Ebenso zentral wie die Wirksamkeit ist die Frage der Reversibilität. Eine internationale Metaanalyse mit über 1500 Teilnehmern zeigte, dass rund 90 % der Männer innerhalb eines Jahres nach Absetzen der Behandlung wieder eine normale Spermatogenese erreichten, nach 2 Jahren lag die Erholungsrate bei nahezu 100 %. Faktoren wie ethnische Herkunft, Ausgangsspermienzahl und die Dauer der Exposition beeinflussten die Geschwindigkeit der Erholung [13].

Die Reversibilität ist nicht nur ein medizinisches Kriterium, sondern eine moralische Bedingung. Eine Verhütungsmethode, die die Aussicht auf spätere Vaterschaft dauerhaft beeinträchtigen könnte, würde dem Prinzip der Autonomie widersprechen und wäre ethisch schwer vertretbar. Ein gesunder Mann sollte sich nur dann einer solchen Intervention aussetzen, wenn gewährleistet ist, dass seine reproduktive Zukunft nicht dauerhaft beschädigt wird. Damit wird die Reversibilität zum Prüfstein der Legitimität männlicher Kontrazeption: Sie entscheidet darüber, ob ein pharmakologisches Verfahren als partnerschaftlich verantwortbar und mit der Würde des Einzelnen vereinbar gelten kann.

Die WHO initiierte 2008 in Kooperation mit dem Contraceptive-Research-and-Development-(CONRAD-)Programm der Universität Arlington, Virginia, USA eine internationale Multicenter-Studie zur hormonellen Kontrazeption beim Mann. Rund 400 Paare wurden mit einer Kombination aus Testosteronundecanoat und Norethisteron-Enantat, appliziert alle 8 Wochen, behandelt. Die Studie bestand aus verschiedenen Phasen: einer Suppressionsphase von bis zu 26 Wochen (d. h. Injektionen in Woche 0, 8, 16 und 24), während der die Männer die Intervention erhielten, einer Wirksamkeitsphase von bis zu 56 Wochen mit fortgesetzten Injektionen, während der die teilnahmeberechtigten Paare dem Risiko einer Schwangerschaft ausgesetzt waren, und einer Erholungsphase (beginnend 8 Wochen nach der letzten Injektion) von bis zu einem Jahr. Die kontrazeptive Wirksamkeit war hoch und übertraf sogar die Effektivität oraler Pillen bei Frauen.

Allerdings entwickelten etwa 10–15 % der Teilnehmer Nebenwirkungen wie depressive Verstimmungen, Libidoverlust, Schwitzen und Gewichtszunahme – überwiegend bedingt durch die antiandrogene Wirkung des Norethisterons. Obwohl diese Effekte medizinisch als moderat einzustufen waren, bewertete die Weltgesundheitsorganisation (WHO) ihre Häufigkeit als nicht akzeptabel und brach die Studie vorzeitig ab [14].

Seitdem richtet sich die Forschung verstärkt auf besser verträgliche Ansätze. Ein transdermales Gel mit Testosteron und Nestoron zeigte bereits in der ersten klinischen Studie eine sehr gute Wirksamkeit bei minimalen Nebenwirkungen [15], setzt jedoch eine hohe Therapietreue voraus [16, 17]. Parallel dazu werden neue orale Steroide entwickelt, die sowohl am Androgen- als auch am Gestagenrezeptor wirken und den First-Pass-Metabolismus umgehen. Besonders sind dies Dimethandrolonundecanoat (DMAU) und 11β-Methyl-19-Nortestosterone-17β-Dodecylcarbonate (11β-MNTDC). Diese erwiesen sich in Phase-I/II-Studien als wirksam und gut verträglich [18, 19].

Der vorzeitige Abbruch der WHO-Studie verweist auf ein Grundproblem: Während Frauen seit Jahrzehnten akzeptieren müssen, dass hormonelle Verhütungsmittel Nebenwirkungen wie Gewichtszunahme, Libidoverlust oder Stimmungsschwankungen mit sich bringen, gelten für Männer offenbar andere Maßstäbe. Diese Asymmetrie wirft die Frage auf, ob die reproduktive Verantwortung gleichberechtigt verteilt wird oder ob das Risiko einseitig weiterhin Frauen zugemutet wird. Im Sinne der Gerechtigkeit und der Achtung beider Geschlechter sollte die Bewertung der Zumutbarkeit von Nebenwirkungen nach einheitlichen Kriterien erfolgen.

## Nichthormonelle Methoden

### Vitamin-A-Antagonisten

Der Retinsäure-Rezeptor Alpha (RARα) hat sich als molekulares Ziel für die nichthormonelle Kontrazeption beim Mann herauskristallisiert. Genetische Knockout-Studien an Mäusen haben gezeigt, dass die Deletion des *Rara*-Gens zu männlicher Sterilität führt, während die allgemeine Gesundheit und das Sexualverhalten erhalten bleiben. Der zugrunde liegende Mechanismus beruht auf der essenziellen Rolle des Retinsäure-Signalwegs in der Differenzierungsphase der Spermatogenese. Eine Unterbrechung dieses Signalwegs stört die Transformation von Spermatogonien zu reifen Spermatiden und resultiert in einer reversiblen Infertilität.

Das Molekül YCT-529 wurde kürzlich als potenter und selektiver RARα-Antagonist entwickelt. Sein Design wurde für eine hohe orale Bioverfügbarkeit, günstige Pharmakokinetik und selektive Bindung an RARα im Vergleich zu anderen Retinsäure-Rezeptor-Isoformen optimiert. In präklinischen Modellen führte die orale Gabe von YCT-529 bei Mäusen innerhalb von 4 Wochen zu einer drastischen Reduktion der Spermienproduktion mit über 99 % kontrazeptiver Wirksamkeit in Paarungsversuchen. Wichtig ist, dass YCT-529 weder die Testosteronspiegel noch allgemeine Gesundheitsparameter wie Körpergewicht, Organhistologie oder Libido beeinflusste.

Nach Absetzen der Behandlung stellte sich die Fertilität bei Mäusen innerhalb von 4–6 Wochen vollständig wieder her. Bei nichtmenschlichen Primaten führte die orale Verabreichung von YCT-529 innerhalb von 2 Wochen zu einer ausgeprägten Suppression der Spermienzahl – erneut ohne systemische Nebenwirkungen. Nach Beendigung der Therapie normalisierte sich die Fertilität innerhalb von 10–15 Wochen vollständig.

YCT-529 befindet sich derzeit in frühen klinischen Studien am Menschen. Eine 28-tägige orale Dosierungsstudie wird aktuell durchgeführt, um Verträglichkeit, Pharmakodynamik und erste kontrazeptive Effekte zu untersuchen. Aufgrund seines nichthormonellen Wirkmechanismus könnte YCT-529 einen Paradigmenwechsel in der männlichen Kontrazeption darstellen [20].

### Lösliche Adenylatzyklase-(sAC-)Inhibitoren

Die lösliche Adenylatzyklase (sAC) ist ein intrazelluläres Enzym, das für die Bildung von zyklischem Adenosinmonophosphat (cAMP) entscheidend ist. In Spermien ist sAC essenziell für die Kapazitation, die Motilität und die Akrosomreaktion –Schlüsselprozesse für eine erfolgreiche Fertilisation.

Die genetische Ausschaltung des *ADCY10*-Gens, das für sAC kodiert, führt sowohl bei Mäusen als auch beim Menschen zu männlicher Infertilität. Dies macht sAC zu einem hochrelevanten, nichthormonellen Ziel für die kontrazeptive Entwicklung. Bemerkenswert ist, dass die Hemmung von sAC die Funktion der Spermien und nicht deren Produktion beeinträchtigt. Dadurch ist sowohl ein schneller Wirkungseintritt als auch eine ebenso schnelle Reversibilität des kontrazeptiven Effekts möglich.

Neuere Studien haben die Machbarkeit einer oralen, schnell wirksamen sAC-Inhibition für eine bedarfsgerechte („on demand“) männliche Kontrazeption demonstriert. Eine einzelne orale Dosis – kurz vor dem Geschlechtsverkehr eingenommen – kann die Spermienmotilität und -funktion vorübergehend hemmen, ohne das Paarungsverhalten oder die systemischen Hormonspiegel zu beeinflussen.

Diese Befunde sind im Bereich der männlichen Kontrazeption beispiellos, da sie erstmals das Konzept einer pharmakologischen, bedarfsgerechten Fertilitätskontrolle mit schneller Reversibilität nach nur einer Dosis einführen. Der Verzicht auf hormonelle Mechanismen reduziert das Risiko systemischer Nebenwirkungen und macht diesen Ansatz besonders attraktiv für intermittierende oder nicht tägliche Anwendungen [21].

Auch wenn die bisherigen Ergebnisse auf präklinischen Tiermodellen beruhen, konzentriert sich die laufende Forschung darauf, diese Moleküle für die Anwendung beim Menschen zu optimieren – insbesondere hinsichtlich Potenz, Wirkdauer und Sicherheitsprofil. Gelingt die Translation, könnten sAC-Inhibitoren eine revolutionäre Option männlicher reproduktiver Autonomie darstellen.

### Testis-spezifische Serin/Threonin-Kinasen (TSSK)

Eine Übersichtsarbeit berichtet, dass die 5 funktionellen TSSK-Kinasen nur in Keimzellen nach der Meiose vorkommen und evolutiv stark konserviert sind. Genetische Knockout-Modelle zeigen, dass diese Kinasen für die männliche Fruchtbarkeit unverzichtbar sind. Wird ihre Funktion gehemmt, kann die Spermatogenese oder die Befruchtung vorübergehend blockiert werden – prinzipiell also reversibel. Eine 2025 veröffentlichte Studie an TSSK1- und TSSK2-Knockout-Mäusen bestätigt, dass der Verlust beider Kinasen zu männlicher Sterilität führt, während weibliche Tiere und andere Organe nicht beeinträchtigt sind. Erste Hemmstoffe mit Pyrrolopyrimidin- oder Pyrimidin-Grundstruktur wurden bereits entwickelt, müssen aber hinsichtlich Selektivität und Spezifität noch weiter optimiert werden [22].

### SLO3-Kaliumkanal

SLO3 ist ein evolutionär stark erhalten gebliebener, spannungsabhängiger Kaliumkanal. Er kommt ausschließlich in den Vorläuferzellen der Spermien, sowohl bei Mäusen als auch beim Menschen, vor. Das zugehörige Gen heißt Kcnu1. Wenn dieses Gen bei männlichen Mäusen ausgeschaltet wird (Knockout), werden die Tiere unfruchtbar, ohne dass andere Organe oder Körperfunktionen beeinträchtigt sind. Deshalb gilt SLO3 als vielversprechendes Ziel für nichthormonelle Verhütungsmittel beim Mann, weil seine Blockade gezielt die Spermienfunktion beeinträchtigt. Es wurde eine große Stoffbibliothek automatisch durchsucht, um Moleküle zu finden, die SLO3 gezielt blockieren. Dabei wurde der selektive Hemmstoff VU0546110 gefunden. Dieses Molekül blockiert SLO3 und verhindert die notwendige Hyperpolarisierung der Spermienmembran, die für die Befruchtungsfähigkeit essenziell ist. Wichtig ist: VU0546110 ist etwa 46-mal selektiver für SLO3 als für den eng verwandten Kanal SLO1, der u. a. im Gehirn und anderen Organen vorkommt. Frühere SLO3-Blocker wie Chinidin waren dagegen unspezifisch und konnten auch SLO1 hemmen, was ein erhebliches Nebenwirkungsrisiko bedeutet hätte [23].

## Perspektiven und Herausforderungen

Der Rückzug großer Pharmaunternehmen seit ca. 2011 aus der Entwicklung männlicher hormoneller Kontrazeptiva hat erhebliche Irritationen ausgelöst. Die Gründe reichen von internen Umstrukturierungen bis hin zu strategischen Neuausrichtungen, die zeitlich mit einer stärkeren regulatorischen Steuerung durch die WHO zusammenfielen.

Damit kam die Weiterentwicklung jedoch nicht zum Stillstand. Stattdessen haben öffentliche Forschungsinstitutionen, internationale Stiftungen und gemeinnützige Organisationen die Führungsrolle übernommen. Besonders im Fokus steht derzeit das transdermale Gel aus Testosteron und Nestoron, das in klinischen Studien eine hohe Wirksamkeit bei guter Verträglichkeit zeigt – auch wenn es täglicher Anwendung und hoher Therapietreue bedarf.

Parallel gewinnen nichthormonelle Strategien an Bedeutung. Hervorzuheben sind Retinsäure-Rezeptor-Antagonisten wie YCT-529, die in Tiermodellen zuverlässig und reversibel wirken und bereits in ersten Humanstudien geprüft werden. Ebenfalls vielversprechend erscheinen Inhibitoren der löslichen Adenylatzyklase (sAC), die eine bedarfsgerechte, präkoitale „On-demand“-Kontrazeption durch temporäre Blockade der Spermienmotilität ermöglichen könnten. Zwar befinden sich diese Ansätze noch in frühen Entwicklungsphasen, doch markieren sie einen konzeptionellen Wandel hin zu hochspezifischen, hormonfreien Verfahren.

Trotz dieser Fortschritte bleibt festzuhalten: Der Einsatz experimenteller Substanzen außerhalb klinischer Studien ist derzeit nicht vertretbar. Ohne robuste Evidenz zu Langzeitsicherheit und Reversibilität birgt ein Off-Label-Gebrauch sowohl rechtliche als auch ethische Risiken. Erst groß angelegte, methodisch strenge Studien können die Grundlage für eine verantwortungsvolle Einführung neuer männlicher Kontrazeptiva schaffen.

Der Rückzug der Industrie wirft die Frage nach ihrer gesellschaftlichen Verantwortung auf. Wer sich aus der Entwicklung zurückzieht, verweist die Last auf öffentliche Einrichtungen und Stiftungen – und macht damit sichtbar, dass die Verhütung nicht nur ein ökonomisches, sondern ein zutiefst ethisches Projekt ist. Die Entscheidung, ob in Forschung investiert wird, sollte nicht allein nach Profitinteressen gefällt werden, sondern muss den Menschen als Zweck an sich in den Mittelpunkt stellen. Denn Freiheit liegt nur in der Anerkennung des anderen: Männer können nur dann gleichberechtigt Verantwortung übernehmen, wenn ihnen sichere Methoden tatsächlich zur Verfügung stehen. Die männliche Kontrazeption verweist auf die existenzielle „Sorge“ des Mitseins – Verantwortung nicht nur für das eigene Leben, sondern auch für das gemeinsame.

## Schlussbetrachtung

Die Entwicklung männlicher Kontrazeptiva befindet sich an einem Wendepunkt. Nach Jahrzehnten der Forschung ist klar: Sowohl hormonelle als auch nichthormonelle Ansätze können zuverlässig, reversibel und prinzipiell sicher wirken. Während hormonelle Kombinationen wie Testosteron/Nestoron oder neuartige orale Steroide (DMAU, 11β-MNTDC) klinisch bereits weit untersucht sind, öffnen innovative nichthormonelle Strategien – etwa Retinsäure-Rezeptor-Antagonisten oder sAC-Inhibitoren – ein neues Kapitel der kontrazeptiven Forschung. Die wissenschaftliche Machbarkeit ist damit zweifelsfrei belegt.

Gleichzeitig wird aber deutlich, dass die Umsetzung nicht allein eine medizinische Frage ist. Sie ist ebenso sehr eine gesellschaftliche und ethische Herausforderung. Die Akzeptanz neuer Methoden hängt nicht nur von ihrer Wirksamkeit ab, sondern von strengen Sicherheitsstandards, der garantierten Reversibilität und einer gerechten Verteilung der Verantwortung zwischen den Geschlechtern. Hier entscheidet sich, ob die männliche Kontrazeption tatsächlich als partnerschaftliche Option wahrgenommen wird – oder ob sie an Skepsis und struktureller Ungleichheit scheitert.

Das Konzept der „Pille für den Mann“ ist somit keineswegs obsolet. Vielmehr befindet es sich in einer Phase strategischer Neuausrichtung und Weiterentwicklung. Es ist in den kommenden Jahren mit weiteren Fortschritten zu rechnen. Insbesondere transdermale und orale Hormonformulierungen sowie neuartige nichthormonelle Methoden könnten bald in den Bereich regulatorischer Zulassung und klinischer Anwendung rücken.

Philosophisch betrachtet führt die Debatte um männliche Verhütung in das Zentrum moderner Ethik. Kontrazeptiva sollten weder Männer noch Frauen als bloße Mittel zur Lastenverteilung dienen dürfen, sondern ihre Autonomie und Würde gleichermaßen achten. Freiheit in der gegenseitigen Anerkennung innerhalb einer Gemeinschaft entsteht, wenn Männer bereit sind, Verantwortung in der Reproduktionsmedizin zu übernehmen, damit verwirklicht sich Gleichberechtigung in der Praxis. Kontrazeption ist Ausdruck existenzieller Sorge – ein bewusstes Gestalten des „Mitseins“, indem Verantwortung für das eigene Leben immer auch Verantwortung für das gemeinsame Leben bedeutet.

Damit wird deutlich: Die „Pille für den Mann“ ist weit mehr als ein medizinisches Projekt. Sie ist Prüfstein für Gleichberechtigung, Ausdruck gelebter Partnerschaft und ein Beitrag zu einer gerechteren Verteilung reproduktiver Verantwortung. Ihr Erfolg wird nicht allein im Labor entschieden, sondern im Spannungsfeld von Wissenschaft, Gesellschaft und Ethik.
